# A randomized, controlled, double-blinded trial on the effects of acellular dermal matrices on the functional assessment and qualitative satisfaction of split-thickness skin grafts

**DOI:** 10.1038/s41598-025-18705-4

**Published:** 2025-09-29

**Authors:** Chae Rim Lee, Woo Shik Jeong, Young Chul Suh, Suk-Ho Moon

**Affiliations:** 1 Department of Plastic and Reconstructive Surgery, Seoul St. Mary’s Hospital, College of Medicine, The Catholic University of Korea, 222, Banpo-daero, Seocho-gu, Seoul, 06591 Republic of Korea; 2 Department of Plastic and Reconstructive Surgery, Asan Medical Center, University of Ulsan College of Medicine, 88, Olympic-ro 43-gil, Songpa-gu, Seoul, 05505 Republic of Korea; 3 Department of Plastic and Reconstructive Surgery, Severance Hospital, Yonsei University College of Medicine, 50-1, Yonsei-ro, Seodaemun-gu, Seoul, 03722 Republic of Korea

**Keywords:** Skin graft, Defect, Reconstructive surgery, Acellular dermal matrix, Diseases, Health care, Medical research

## Abstract

Acellular dermal matrix (ADM) has been widely used in skin grafting to provide a dermal layer for facilitating graft take and mature scarring. This randomized controlled, double-blinded superiority study aimed to quantitatively assess the effects of this technique on the resulting graft sites via functional measurements including elasticity, humidification, transepidermal water loss (TEWL), pigmentation, and erythema at 6 months after surgery. A survey was performed to evaluate the satisfaction levels of both patients and surgeons. We considered patients with full-thickness skin defects for whom split-thickness skin grafts (STSGs) were used. By random assignment, they were allocated to the ADM group or the STSG-only group. The ADM group received STSG with a ADM layer on the defect, whereas the STSG-only group was reconstructed with only STSG. At 6 months after surgery, patients were evaluated for elasticity, humidification, TEWL, erythema, and pigmentation. Defect areas before and 2 weeks after surgery were traced and measured. Patients and surgeons rated the graft sites as excellent, good, fair or poor at 6 months after the operation. Among the 89 patients who were screened, 54 patients were enrolled in this study. Compared with those in the STSG-only group, defects that were reconstructed with STSG and ADM resulted in significantly greater elasticity, lower TEWL, and less erythema. There were no significant differences in humidification or pigmentation. The ADM co-grafted with STSGs was associated with greater satisfaction among surgeons, whereas patient satisfaction was not significantly different between the two groups. Co-grafting ADM with STSG in full-thickness wounds can lead to favorable wound healing in terms of elasticity, TEWL, and erythema. This approach also has a higher satisfaction rate for surgeons.

**Registry:** Clinical Research Information Service (https://cris.nih.go.kr), Registration number: KCT0007040, Registration date: 24/02/2022.

**Supplementary Information:**

The online version contains supplementary material available at 10.1038/s41598-025-18705-4.

## Introduction

The acellular dermal matrix (ADM) is a versatile tool in the fields of reconstructive surgery and wound healing. It is an allogenic or xenogenic immunologically inert processed layer of dermis that can be utilized as an extracellular matrix that provides scaffolding and facilitating wound healing^1–3^. It is widely used in various areas, including breast reconstructions^4^, head and neck surgeries^5^, lower extremity reconstructions^6,7^, burn wounds, and skin grafts^8^. While synthetic skin replacements are under exploration, ADM is a convenient and relatively affordable source of soft tissue that could be otherwise difficult to procure^9^.

One of the main functions of ADM is providing a protective and incorporable layer for wound coverage. For shallow and expansive wounds, finding a source of dermal replacement with limited donor sites is challenging. For deeper wounds, protecting the underlying structures, such as tendons, nerves, muscles, and bones, and facilitating skin graft integration is important^8,10^. In both cases, split-thickness skin grafts (STSGs) constitute a central method of treatment for large, shallow defects and are often cografted with ADMs^11^.

While ADM is a widely used tool, its effectiveness on STSG outcomes has not been quantitatively analyzed. Many studies have affirmed that ADM is beneficial to reconstruction with skin grafts, but few have objectively inspected the healed tissues in terms of measured parameters. Among many parameters, we have chosen to inspect elasticity, skin humidification, transepidermal water loss (TEWL), erythema, and pigmentation in order to assess the biomechanical properties of the skin barrier^12–15^.

This randomized, double-blinded, superiority trial investigates the effect of ADM on STSG healing by comparing tissue characteristics, including elasticity, erythema, scar humidification, transepidermal water loss, and the melanin index. In addition, patient and surgeon satisfaction was assessed using subjective surveys.

## Method

### Study design

This study was a randomized, double-blinded, superiority trial that was conducted at Seoul St. Mary’s Hospital, Yonsei Severance Hospital, and Seoul Asan Hospital to evaluate the efficacy of combining STSG with human ADM (L&C Bio, Seongnam-si, Gyeonggi-do, South Korea) in the reconstruction of full-thickness skin defects between 2022 and 2024.

Trial protocol and data are available in supplementary files.

### Ethical review

This study was reviewed and approved by the Institutional Review Boards (IRBs) of Seoul St. Mary’s Hospital, Asan Medical Center, and Severance Hospital before and throughout the experiment. Any significant modifications related to the study were subject to IRB approval, and all results have been registered in the Clinical Research Information Service on 24/02/22 (https://cris.nih.go.kr, Registration number KCT0007040). It was conducted in compliance with the principles of the International Conference on Harmonization (ICH) Guidelines and the Declaration of Helsinki. This study was carried out in accordance with the Korean Good Clinical Practice (KGCP) guidelines and relevant regulations, with due consideration for the rights and safety of the study participants.

As a postmarketing clinical study of Megaderm Plus (L&C Bio, Seongnam-si, Gyeonggi-do, South Korea) performed within the approved indications, it is not subject to approval by the Ministry of Food and Drug Safety (MFDS).

### Study population

Patients between 19 and 70 years of age with full-thickness skin defects requiring reconstruction were provided with informed consent to participate in the clinical trial. Defects were acute, uninfected wounds on flat surfaces that were not affected by joint movements. Target enrollment was 84 subjects, with 42 per group, accounting for 20% dropout. Patients were excluded if they were pregnant, nursing, or had a history of potentially relevant or unsuitable conditions that could interfere with wound healing.

### Randomization and maintaining blindness

Each enrolled patient was assigned a screening number and screened per the eligibility criteria. After completing the screening process, each eligible patient was randomly assigned a 3-digit number and assigned to either the experimental (ADM and STSG) or control group (STSG only) according to a computer-generated randomization table. This allotment was performed via the block randomization method, in which each patient was stratified to each institution so that the control group and experimental groups could be balanced. The three-digit randomization number was used as the patient identification code during the experiment.

This study maintained double-blindness by establishing an independent investigator to measure and analyze the functional assessments. Because the investigator was not involved in the surgical process, he or she was able to make objective measurements without bias. The operator was aware of the patient allocation during the surgery but was blinded during the satisfaction survey.

### Safety analysis and discontinuation of trial

Adverse events were assessed at every visit after the operation and were classified according to severity; mild, if it does not interfere with normal daily activities, moderate if it causes noticeable discomfort affecting normal daily activities, or severe if it prevents normal daily activities. If unexpected surgical complications occur in more than 30% of participants, the trial was to be discontinued.

### Surgical procedure and postoperative care

**Fig. 1:**
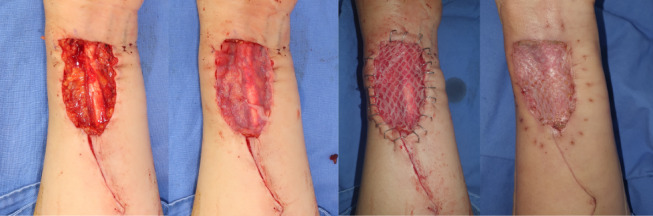
Clinical photos of reconstructing full-thickness defects with ADM and STSG. (**a**) A full-thickness surgical defect requires reconstruction. (**b**) ADM is trimmed and placed on the defect. (**c**) Meshed STSG is placed and fixed with skin stapler. (**d**) STSG was well-taken at 2 weeks post-operative.

**Fig. 2:**
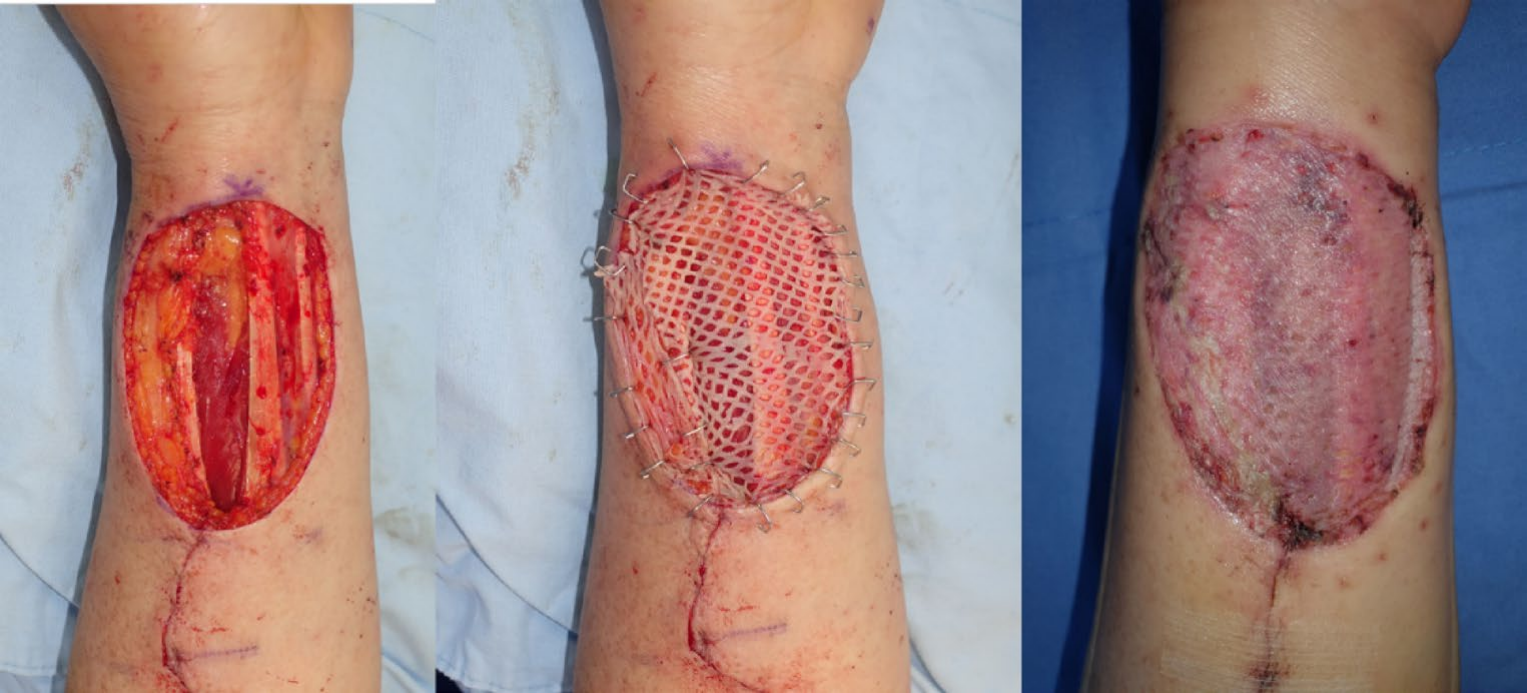
Clinical photos of reconstructing full-thickness defects with STSG only. (**a**) A full-thickness surgical defect requires reconstruction. (**b**) Meshed STSG is placed and fixed with skin stapler. (**c**) STSG was well-taken at 2 weeks post-operative.

Before skin grafting, all wounds were debrided, thoroughly irrigated, and cauterized for bleeding. When the full-thickness skin defect was prepared for skin grafting, it was traced for area measurements before grafting. Split-thickness skin grafts were harvested from either thigh with a pneumatic dermatome at 0.002 mm thickness. The ADM group received a combination of cadaveric ADM (MegadermPlus, L&C Bio, Seongnam-si, Gyeonggi-do, South Korea) and STSG, whereas the control group received STSG alone. For the ADM group, human ADM was placed on the defect and was trimmed to fit before applying the STSG (Fig. 1). For the control group, skin grafts was placed directly on the defects. STSGs were meshed, trimmed to the defect size, and fixed on the wounds with skin stapler (Fig. 2). The wounds were dressed under negative pressure wound therapy (NPWT). When applying NPWT (Curasys, CG Bio, South Korea), a layer of gel matrix dressing (UrgoTul, Urgo medical, France) was placed on the skin graft before applying the sponge and were then vacuum sealed with film at continuous negative pressure of 120 mmHg. The dressing was removed after 3 to 5 days, followed by gauze or foam dressing, which was changed every two days. Dressings were discontinued once the wounds healed and no longer showed exudates. A skin moisturizer provided by the investigator was applied for at least 3 months, except for the 7 days preceding the follow-up visits. The moisturizer (Bythedoctor Ato lotion, L&C Bio, South Korea) contained hyaluonic acid, ceramide, and lecithin and was used as an emollient barrier to promote skin remodeling and healthy scar formation to the grafted sites. It was discontinued before data collection to prevent its impact on data measurements.

### Postoperative follow-ups and evaluations

Follow-up observations were performed at 2 weeks, 3 months, 6 months, and 12 months after surgery. All assessments and measurements were done by a blinded investigator who had not participated in the randomization process or the surgical procedures. At each visit, the patients’ vital signs were measured, photographs of their surgical sites were taken, and any adverse effects were observed. At the 2 week visit, pictures of graft sites were taken and areas of successfully healed grafts were measured by ImageJ program. At the 6-month visit, the patients were analyzed for their functional skin assessments and satisfaction.

Primary outcome was skin elasticity at 6 months after surgery. Secondary outcomes included other functional skin assessments at 6 months after surgery, such as scar humidification, TEWL, melanin index, and erythema index. Elasticity was measured with a Cutometer MPA-580 (Courage & Khazaka Electronic GmbH, Germany) under 500 mbar vacuum. Scar humidification was measured with a Corneometer (Courage & Khazaka Electronic GmbH, Germany) and a CM825 probe. TEWL was measured with a Tewameter (Courage & Khazaka Electronic GmbH, Germany) and TM300 probe. The melanin index and erythema index were measured with a Mexameter (Courage & Khazaka Electronic GmbH, Germany) with an MX18 probe.

The graft areas were traced during surgery before grafting and 2 weeks after surgery. Tracings were then scanned, and the area was measured via the ImageJ program. A blinded satisfaction survey was performed at 6 months after surgery by both patients and surgeons, who rated their resulting skin graft sites as excellent, good, fair, or poor.

### Statistical analysis

The functional assessment of skin elasticity, humidification, transepidermal water loss (TEWL), the melanin index, and erythema at 6 months postsurgery was performed. Group comparisons were performed via either the independent two-sample t test or Wilcoxon rank-sum test, which is based on normality assumptions per the Shapiro‒Wilk test. If the patient was missing any data, the data was excluded from analysis.

The satisfaction scores from both the investigators and the participants are presented as frequencies. Comparisons between groups were made via Fisher’s exact test if there were more than 5 cells with expected counts of less than 5.

The graft rate at 2 weeks postsurgery was calculated by comparing the engrafted area to the initial treated area for each treatment group. Group comparisons were performed via the Mann‒Whitney U test after testing for a normal distribution via the Shapiro‒Wilk test.

**Fig. 3:**
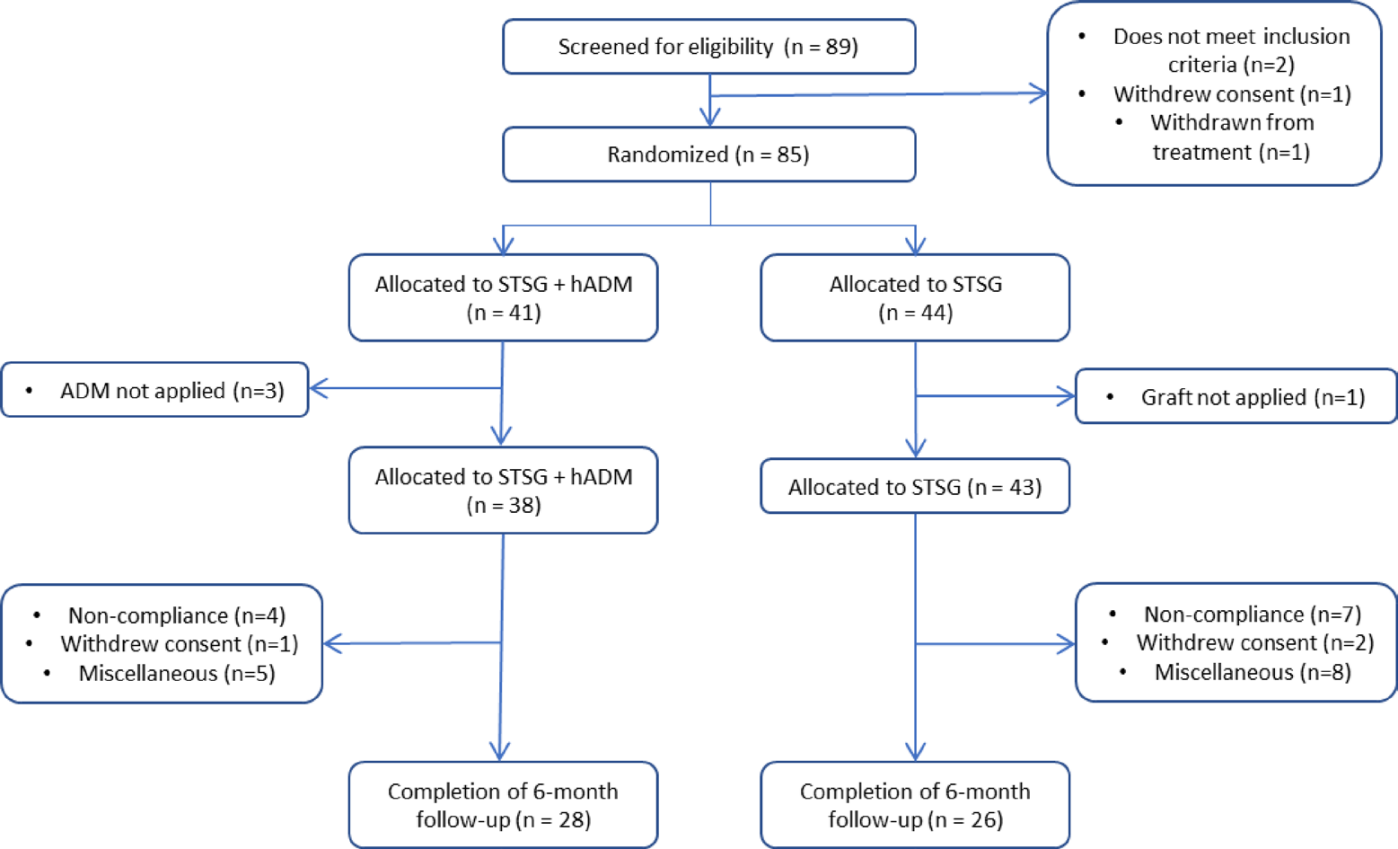
Patient enrollment, randomization, and follow-up.

## Results

Eighty-nine patients were screened for eligibility, and 85 patients who met the eligibility criteria were randomized into ADM-only and STSG-only groups. Forty-one and 44 patients were allocated to the ADM-only and STSG-only groups, respectively. During the surgical procedures, a different surgical method was selected for 3 patients in the ADM group and 1 patient in the STSG-only group. During postoperative follow-up, 10 and 17 patients, respectively, were withdrawn from the study because of noncompliance (4 and 7 patients, respectively), withdrawal of consent (1 and 2 patients, respectively), and otherwise, miscellaneous reasons (5 and 8 patients, respectively). As a result, a total of 54 patients were enrolled per protocol this study, with 28 in the ADM group and 26 in the STSG only group (Fig. 3).

Both groups did not differ significantly in terms of demographic factors, such as age, sex, previous medical history, defect location, or cause of defects. The mean ages of the ADM and STSG-only groups were 56.6 and 57.8 years, respectively. Nearly all patients had previous illnesses, including soft tissue malignancies. Most of the defects were free flap donor sites (92.9% and 88.5%, respectively) located at thigh and forearm areas. Other defects were caused by other means such as trauma, burn, fasciotomy, and tumor excision. All defects were clean, acute wounds located on flat surfaces not associated with joint movements (Table 1).

**Fig. 4:**
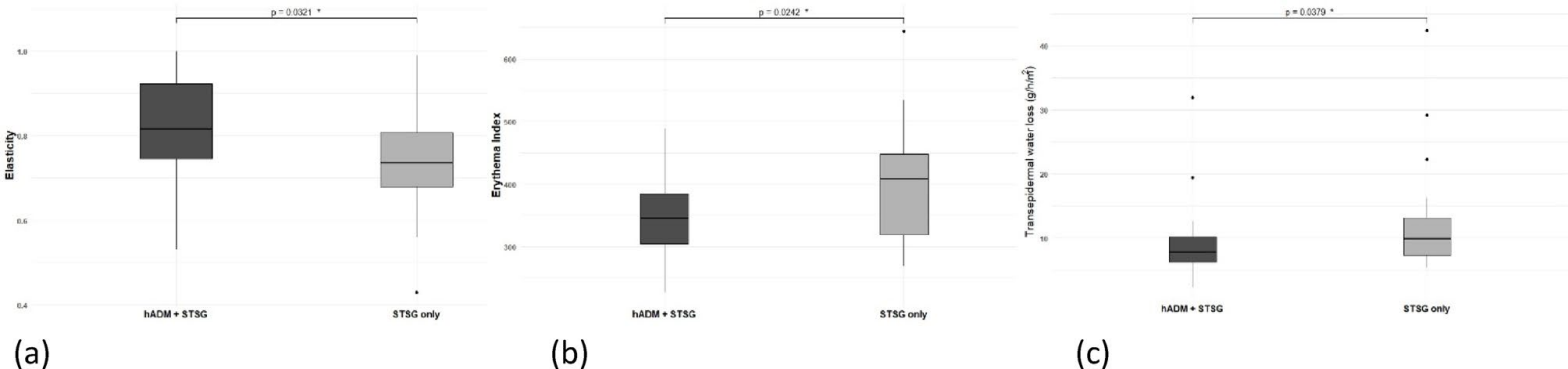
Comparison between STSG with and without ADM. (**a**) Elasticity, (**b**) Erythema, (**c**) Transepidermal water loss (*: statistically significant).

Skin assessment at 6 months after surgery revealed elasticity, humidification, transepidermal water loss (TEWL, g/hr/m^2^), pigmentation, and erythema. Elasticity index, the primary outcome, was significantly greater in the ADM group (0.82 ± 0.13) than in the STSG-only group (0.74 ± 0.12), with a p-value of 0.032. Secondary outcomes differed by category. Humidification was slightly greater in the STSG group than in the ADM group, at 32.5 ± 12.6 and 28.2 ± 14.1, respectively, but the difference was not statistically significant. TEWL was greater in the STSG-only group (12.29 ± 8.24) than in the ADM group (8.99 ± 5.57) with a p-value of 0.038. Pigmentation, as measured by the melanin index, was 269.2 ± 130.2 and 270.2 ± 144.8 for the ADM group and the STSG group, respectively, and the difference was not statistically significant. Erythema index was significantly greater in the STSG group (399.8 ± 90.9) than in the ADM group (350.4 ± 64.02), with a p value of 0.024. (Fig. 4; Table 2)

The graft area was measured before grafting and 2 weeks after surgery. The mean defect areas were 34.51 ± 29.81 cm^2^ and 46.76 ± 46.59 cm^2^ in the ADM and STSG-only groups respectively, with no significant differences. The area of healed grafts at 2 weeks was 33.57 ± 29.84 cm^2^ (95.4 ± 9.2%) for the ADM group and 46.37 ± 46.14 cm^2^ (98.8 ± 2.15%) for the STSG-only group. More than 95% of the grafts in both groups were successfully engrafted by 2 weeks after surgery, and there were no significant differences between the groups.

**Fig. 5:**
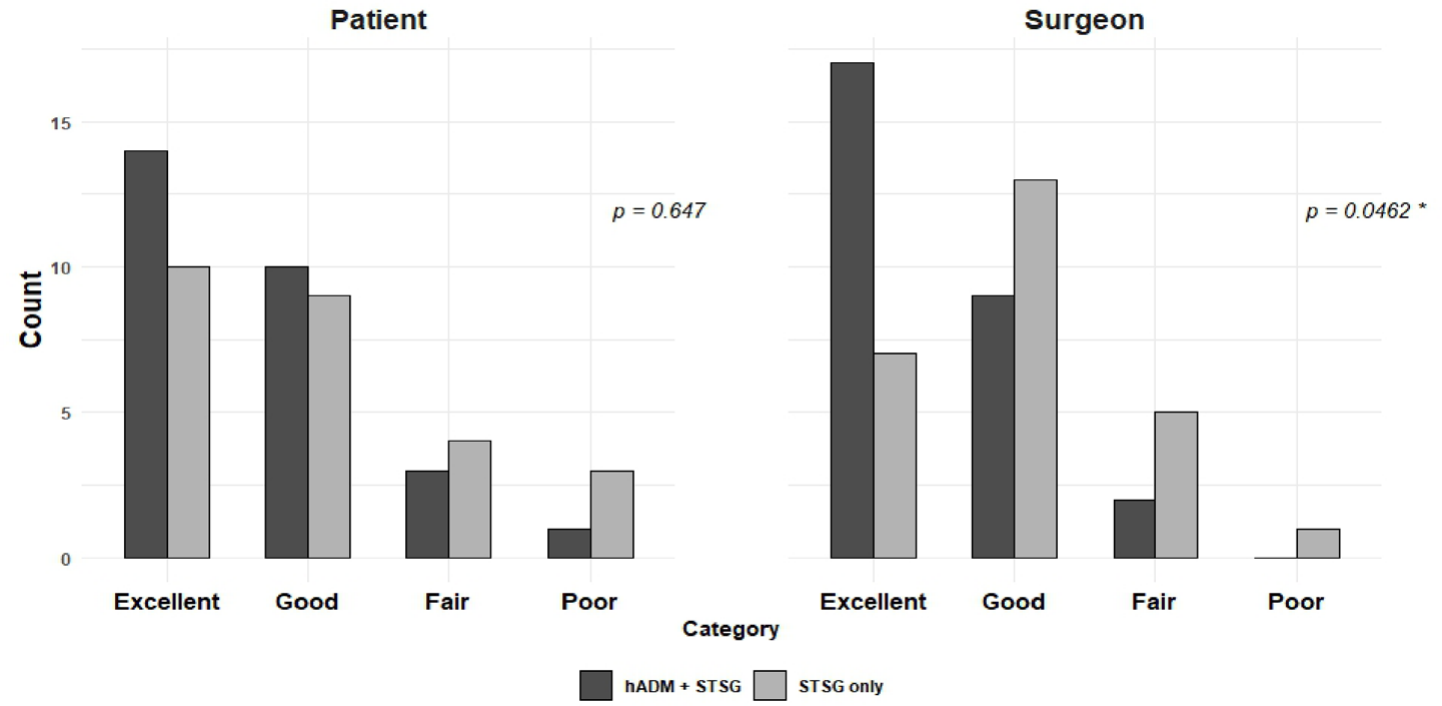
Results of patient and surgeon satisfaction survey (*: statistically significant).

Patient and surgeon satisfaction analysis was performed at 6 months after surgery. (Table 3) Surveys were double-blinded and categorized into 4 categories: excellent, good, fair, and poor. The surgeons’ responses revealed that the ADM group was significantly more favorable with a p-value of 0.046. A total of 96.8% of the surgeons reported that the ADM co-grafted STSG sites were excellent or good, whereas only 2 (7.14%) thought that they were fair. No surgeon reported that the STSGs co-grafted with ADMs was poor. The majority, 89.5% and 73.1%, of the patients in the ADM group and the STSG-only group, respectively, reported favorable answers of excellent or good, and 3.6% and 11.5%, respectively, reported that the graft site quality was poor. There was no significant difference between the ADM group and the STSG-only group (Fig. 5).

Nine adverse events were reported among 6 subjects (21.4%) in the test group and 37 among 10 subjects (38.5%) in the control group, but none were relevant to the procedure. In the test group, the adverse events were operation site pain, hypertension, vertigo, hemoptysis, arm swelling, arm pain, soft tissue abscess, and cancer metastasis. The control group reported symptoms such as vomiting, diarrhea, pruritus, and facial edema, but none were relevant to the procedure. There was no statistically significant difference between the two groups (*p* = 0.35). There was no graft failure or complications related to wound healing.

**Table 1:** Demographics.

Variable	ADM + STSG (%)	STSG only (%)	*p* value
Number of patients	28	26	
Age (mean ± SD)	56.6 ± 12.0	57.8 ± 13.4	0.730^1^
Sex			0.155^2^
Male	16 (57.1)	20 (76.9)	
Female	12 (42.9)	6 (23.1)	
Previous illness			1.000^2^
Yes	26	25	
No	2	1	
Defect location			0.345^2^
Ankle	1 (3.6)	0 (0.00)	
Arm	3 (10.7)	1 (3.8)	
Foot	0 (0.00)	1 (3.8)	
Forearm	6 (21.4)	3 (11.5)	
Lower leg	3 (10.7)	1 (3.8)	
Thigh	15 (53.6)	20 (76.9)	
Cause of defect			0.731^2^
Free flap donor site	26 (92.9)	23 (88.5)	
Trauma	1 (3.6)	1 (3.8)	
Chemical burn	0 (0.00)	1 (3.8)	
Fasciotomy	1 (3.6)	0 (0.00)	
Tumor excision	0 (0.00)	1 (3.8)	

^1^Independent two-sample t test, ^2^Fisher’s exact test, SD : standard deviation.

**Table 2:** Results of skin assessment at 6 months after surgery.

Variable	ADM + STSG	STSG only	*p* value
Number of patients	28	26	
Elasticity index	0.82 ± 0.13	0.74 ± 0.12	0.032* ^1^
Humidification index	28.2 ± 14.1	32.5 ± 12.6	0.25 ^1^
Transepidermal water loss (g/hr/m^2^)	8.99 ± 5.57	12.29 ± 8.24	0.038 * ^2^
Melanin index	269.2 ± 130.2	270.2 ± 144.8	0.95 ^2^
Erythema index	350.4 ± 64.02	399.8 ± 90.9	0.024 * ^1^

*Statistically significant/^1^Independent two sample t test, ^2^Mann-Whitney U test.

**Table 3:** Patient and physician satisfaction analysis at 6 months after surgery.

	Patient		Surgeon	
Variable	ADM + STSG	STSG only	ADM + STSG	STSG only
Excellent (%)	14 (53.8)	10 (38.5)	17 (64.7)	7 (26.9)
Good (%)	10 (35.7)	9 (34.6)	9 (32.1)	13 (50)
Fair (%)	3 (10.7)	4 (15.4)	2 (7.14)	5 (19.2)
Poor (%)	1 (3.57)	3 (11.5)	0 (0)	1 (3.85)
Total	28	26	28	26
p value	0.617^1^		0.0462* ^1^	

^1^Fisher’s exact test, * : statistically significant.

## Discussion

Skin grafting is a useful reconstructive method that is versatile for most defects, but it is limited by donor site availability^16^ and its thinness^17,18^. To compensate for these disadvantages, various combinations of allogenous and autogenous grafts have been explored^19,20^, finally giving way to ADMs in the 1990s. ADMs have been introduced as an immunologically inert form of allogenous or xenogenous skin layer^21,22^ for coverage of expansive full-thickness skin injuries.

Various forms of ADMs have been explored, ranging from allografts and xenografts to artificial matrices, that can facilitate tissue growth with minimal immunologic reactions or scarring^23,24^. Allogenic ADMs are obtained from human cadaveric skin, treated to minimize immunologic triggers. Xenogenic ADMs are most frequently prepared from bovine or porcine skin and are economically favorable. Some animal model studies have shown, however, that xenografts are associated with increased cell-mediated immune reactions^25,26^ and poor wound healing^26,27^.

In the clinical setting, both allogenic and xenogenic ADMs are actively used in reconstructive surgery. They are mainstay in breast reconstruction for natural aesthetic outcomes and to prevent capsular contracture in breast^28,29^. Cosmetic surgery also uses ADMs as a source of augmentation that can minimize foreign body reactions^30^, since inflammation can cause significant complications. All areas of reconstructions, including head and neck, genitourinary, and lower extremities, are actively utilizing ADM as an additional source of soft tissue. However, ADMs are costly^1,31,32^, requiring both patient and surgeon to weigh the pros and cons of this reconstructive tool. While there are several studies that warrant the use of ADM resulting in an overall reduction in time and cost^31,33^, some assert that it does pose a financial burden on patients^31,34^ and to be wary of industrial fundings that may be a cause of this surge of ADM in the clinical and academic settings^32^.

Especially in split-thickness skin grafts, supplementation with ADM can provide a layer of acellular extracellular matrix (ECM), a kind of biocompatible scaffold for tissue ingrowth, and mimic the physiologic dermis layer that STSGs lack. Full thickness skin grafts (FTSGs), skin grafts that include the dermis layer, are often considered as an alternative, but they are limited by donor site morbidity in expansive defect coverage. ADM acts as a supplementary dermis in STSGs, and is known to facilitate wound healing by providing a template^3,35^ for fibroblasts to infiltrate and provide collagen for wound coverage^36^. Owing to its compositional similarity to the dermis^36,37^, it can assist in the functions of the skin barrier, such as durability^37^ and protection against loss of humidification, as well as deterrence of hyperpigmented and immature scarring. Some studies^6,9^ have used ADM as a single treatment option to promote wound healing without autologous tissue transfer.

This study aimed to assess the function and aesthetics of healed defects to objectively analyze the effect of ADM on STSG by quantitative parameters and surgeon-/patient-reported quality outcomes. Functional measures were reported by elasticity, a parameter reflecting skin plasticity and durability^37^. TEWL and skin humification measured skin barrier integrity^38^, reflecting the ability of stratum corneum’s retention of the body’s moisture held from the external environment^13,14^. For aesthetic considerations, we measured erythema and pigmentation, with erythema an important predictor of clinical symptoms of scar formation^39^, such as pruritus and pain.

At 6 months after surgery, the primary outcome showed that skin elasticity was higher in the STSG and ADM group. Lee et al.^40^ reported that the elasticity of the human ADM co-grafted to STSG was similar to that of adjacent normal skin. Because ADM is composed of type I collagen^35^, it can be expected that applying ADM with STSG can increase skin elasticity. Other studies have recently explored the modification of this scaffold to augment its elasticity^37,41^.

For secondary outcomes, we measured other variables to attest for the biomechanical properties of grafted skin. Our experiment revealed that skin grafts with ADM presented a lower TEWL, confirming that ADM can act as a functional assistance against moisture loss. Other studies^40^ also reported similar results, where human or bovine ADM co-grafted STSGs presented lower TEWL than did the STSG-only group. Although there was no significant difference in scar humidification between the two groups, the resistance to water loss was greater in the ADM group than in the control group, indicating that the skin barrier function improved.

ADM co-grafted STSGs were significantly less erythematous than STSG-only group, indicating that ADM co-grafted with STSGs may reduce the possibility of future scar symptoms. Pigmentation was not significantly different between the groups while other studies^40^ explored the differences of pigmentations in allogenic and xenogenic ADMs.

According to our satisfaction survey, ADM was significantly favored by surgeons, while there was no significant difference among patients. Given that this is a double-blinded study, STSG sites co-grafted with ADM were rated more favorable to the professional eye. It also shows that there was no outstanding downside in the use of ADM compared to STSG only reconstructions.

Limitations of this study are underscored by the shortness of the follow-up period. Evaluations of functional assessments would benefit from a longer observation to see the changes made with skin remodeling. Satisfaction survey could be done in more detail to analyze the reasons behind the responses.

## Conclusion

This double-blinded controlled study objectively shows that human ADM can enhance the skin grafts in elasticity, TEWL, and erythema. Subjective survey showed that surgeons viewed ADM co-grafted skin as more favorable to the surgeons and that patients did not complain of significant differences. We corroborate that ADM is a beneficial tool in STSG and suggest a long-term study for its impact on skin remodeling.

## Supplementary Information

Below is the link to the electronic supplementary material.

Supplementary Material 1

## Data Availability

The datasets generated and/or analysed during the current study are available in the CRIS repository (cris.nih.go.kr) under Registration number KCT0007040, registraction date of 24/02/2022. The datasets used and/or analysed during the current study available from the corresponding author on reasonable request. All data generated or analysed during this study along with the trial protocol are included in this published article and its supplementary information files.
